# Gastrointestinal Perforation With an Intraluminal Stent and Bevacizumab use in Advanced Metastatic Colorectal Cancer

**DOI:** 10.7759/cureus.12831

**Published:** 2021-01-21

**Authors:** Nouf Akeel, Wafaa A Toonsi

**Affiliations:** 1 Surgery, King Abdulaziz University Faculty of Medicine, Jeddah, SAU

**Keywords:** gastrointestinal perforation, intraluminal stent, bevacizumab, metastatic cancer, colorectal cancer, case report

## Abstract

Intestinal obstruction is a common acute presentation of advanced rectal cancer, which could be managed with surgical or non-surgical techniques including metallic stenting. Bevacizumab has been gaining popularity in the treatment of advanced colorectal cancer (CRC) in combination with different chemotherapeutic agents, to improve the overall survival rate; however, data regarding the adverse effects of bevacizumab in combination with other treatment modalities have been insufficient. Herein, we present a case of a 37-year-old man diagnosed with advanced rectal cancer with concurrent liver and lung metastases. He was started on Xelox (capecitabine plus oxaliplatin) chemotherapy in combination with bevacizumab for palliative care. He developed an episode of bowel obstruction, which was managed with emergent placement of a metallic stent. Soon after that, the patient presented emergently with signs and symptoms of intestinal perforation. He underwent emergent surgical intervention with stoma creation and a complicated hospital course. Despite the oncological benefits of bevacizumab for treating metastatic CRC, complications can occur resulting in a devastating outcome, with intestinal perforation being the most serious rare complication.

## Introduction

Colorectal cancer (CRC) is considered the third most common malignancy worldwide, affecting 1.4 million humans each year. Its wide distribution and natural history make it the fourth leading cause of cancer deaths globally [1]. 

Rectal cancer represents 35% of the total incidence of CRC in the European Union [2]. Presentations range from asymptomatic, incidental findings to locally advanced and metastatic disease, where complications have already occurred. Studies found that 25% of patients present late in stage 4, 85% of whom initially present with intestinal obstruction that manifests as vomiting, obstipation, or abdominal distention. Management of advanced cases includes a variety of surgical and non-surgical options, which may either be curative or palliative. However, poor overall survival and a survival deficit persist for up to one year after surgical intervention as candidates are usually older and have other comorbidities [3].

One of the acceptable surgical therapeutic options to relieve symptoms of obstruction related to advanced CRC in emergency situations is the Hartmann procedure with colostomy creation. Although this approach has been the gold standard for managing emergency situations over the past two decades, its complications are devastating [4]. Self-expanding metallic stents (SEMS) have gained popularity in the management of distal malignant obstruction, either for palliation or as a bridge to surgery for resectable tumors, allowing relief of the obstruction without stoma formation [5]. Although SEMS have success rates exceeding 90% and mortality rates as low as 1%, the possibility of developing [6] a life-threatening perforation raises the mortality rates to approximately 20%-30% [7], despite an overall perforation incidence of 4% [6].

Bevacizumab (Avastin®), a monoclonal antibody that blocks vascular endothelial growth factor, is an important mediator of tumor angiogenesis [8]. Studies have demonstrated that bevacizumab significantly improves the overall survival in patients with advanced metastatic CRC [9].

A study of patients with advanced CRC who were treated with bevacizumab in combination with chemotherapy and surgical interventions with a total of three years surveillance found that, for all patients who were treated with bevacizumab, most adverse effects were hematological (neutropenia and leukopenia). No severe bevacizumab-related toxicities were noted in this study, including bleeding, gastric-intestinal perforation, and thromboembolism. Despite its oncological benefits, bevacizumab can result in rare complications such as bowel perforation, a life-threatening condition with poor survival and devastating outcomes [10].

## Case presentation

**Figure 1:**
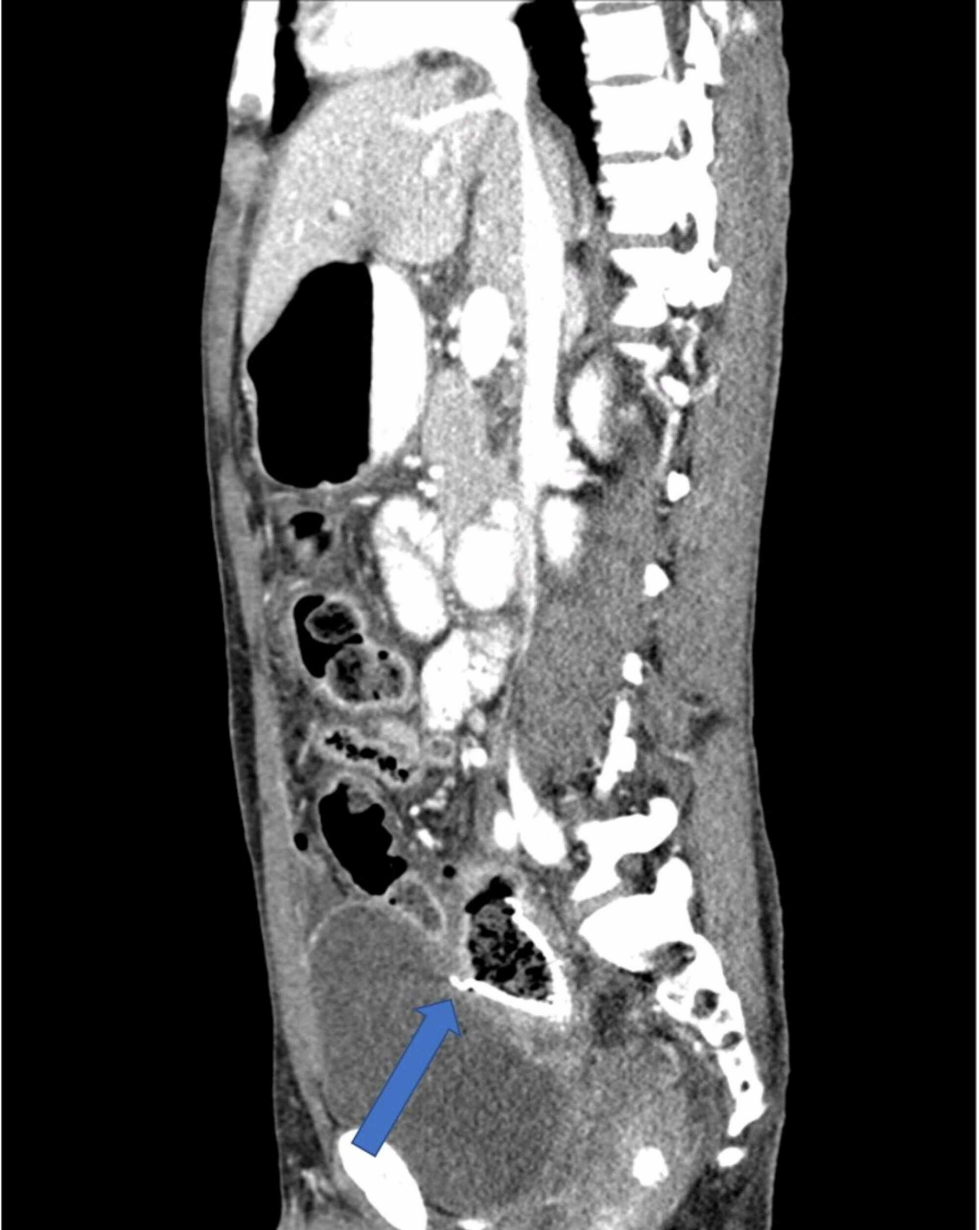
CT scan of the abdomen demonstrating the rectal stent in its place but intending the anterior rectal wall; note the free air in the abdomen (sagittal view)

**Figure 2:**
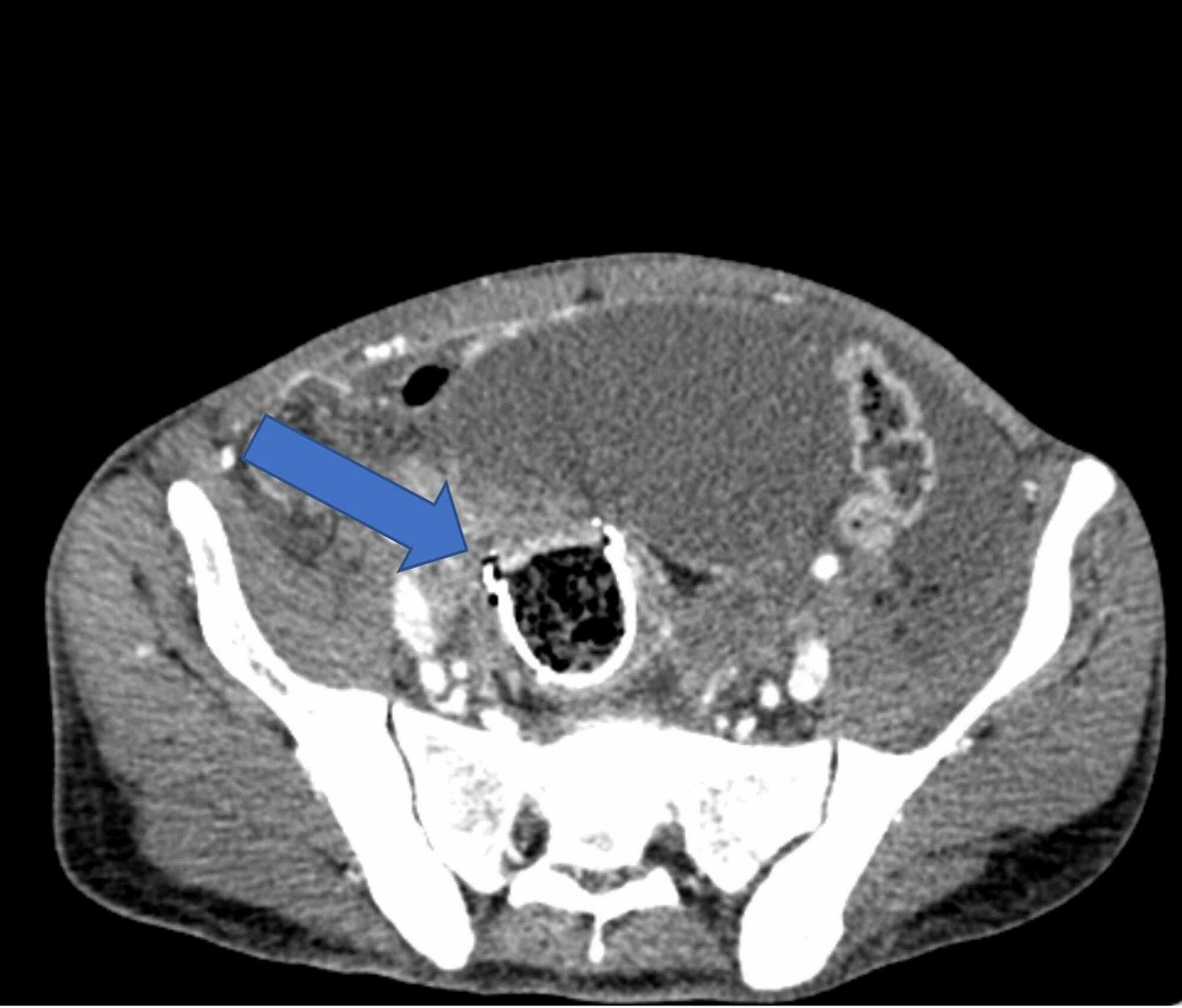
CT scan of the abdomen demonstrating the rectal stent in its place but intending the anterior rectal wall; note the free air in the abdomen (axial view)

**Figure 3:**
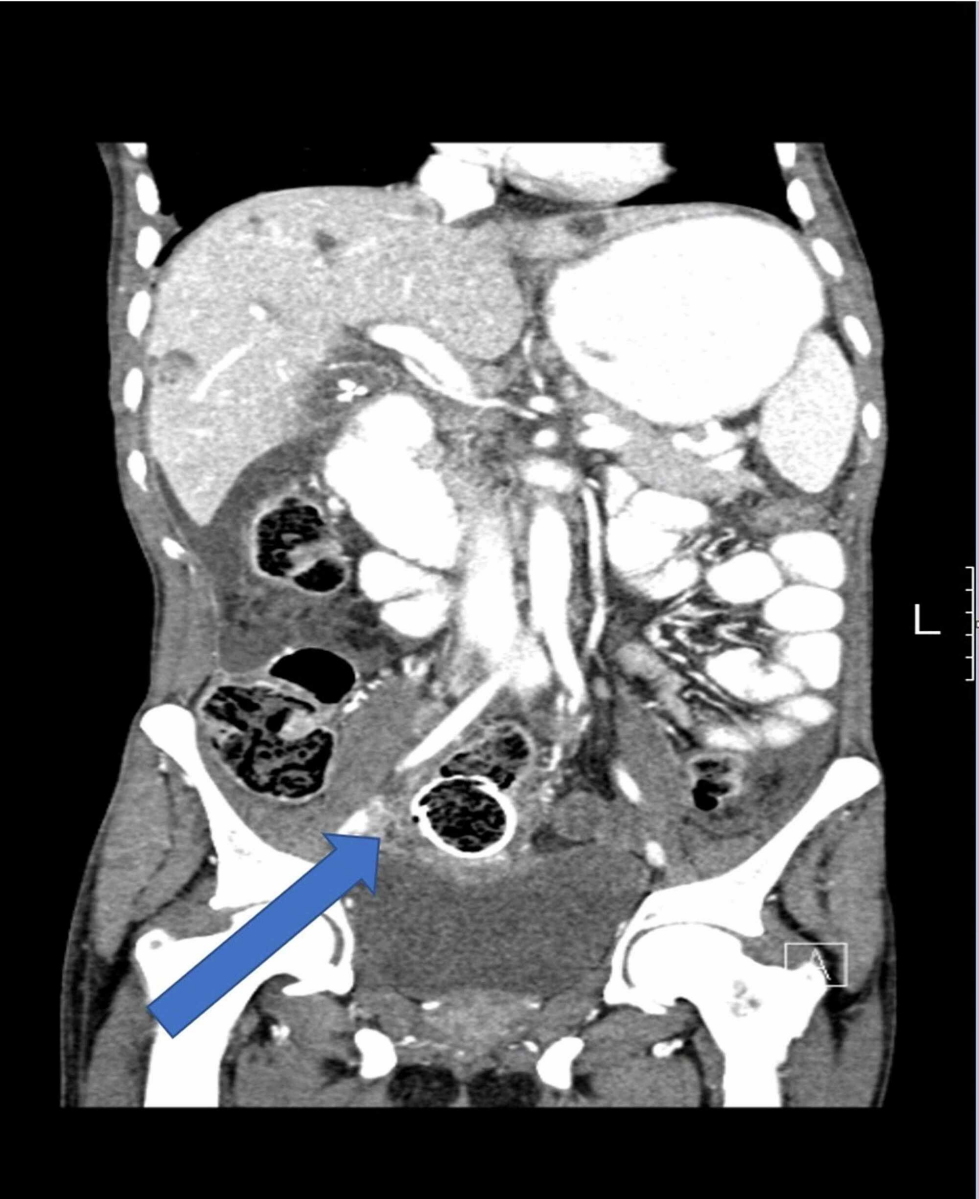
CT scan of the abdomen demonstrating the rectal stent in its place but intending the anterior rectal wall; note the free air in the abdomen (coronal view)

A 37-year-old man was diagnosed with advanced rectosigmoid adenocarcinoma with concurrent lung and liver metastasis and received palliative Xelox (capecitabine plus oxaliplatin) chemotherapy in combination with bevacizumab. He presented to the emergency department at King Abdelaziz Hospital in the Kingdom of Saudi Arabia, with symptoms suggestive of intestinal obstruction. He was managed with placement of a metal stent and discharged in a good condition. Three weeks later, a few days after he finished his 7th and last cycle of chemotherapy, he presented to the emergency department again complaining of a two-day history of abdominal pain. Physical examination found a rigid peritonitic abdomen, tachycardia of 110, and blood pressure of 110/60. His lab results were unremarkable, and initial erect and supine abdominal radiographs showed bowel loops distended within normal limits, no air-fluid level, and no free air. His abdominal and pelvic CT scans with intravenous and oral contrast revealed a stent in its proper position with the superior anterior part indenting the anterior rectal wall, which led us to suspect perforation (Figures 1-3). Free fluid with free air was also noted in the sub-hepatic and right pelvic regions. 

**Figure 4:**
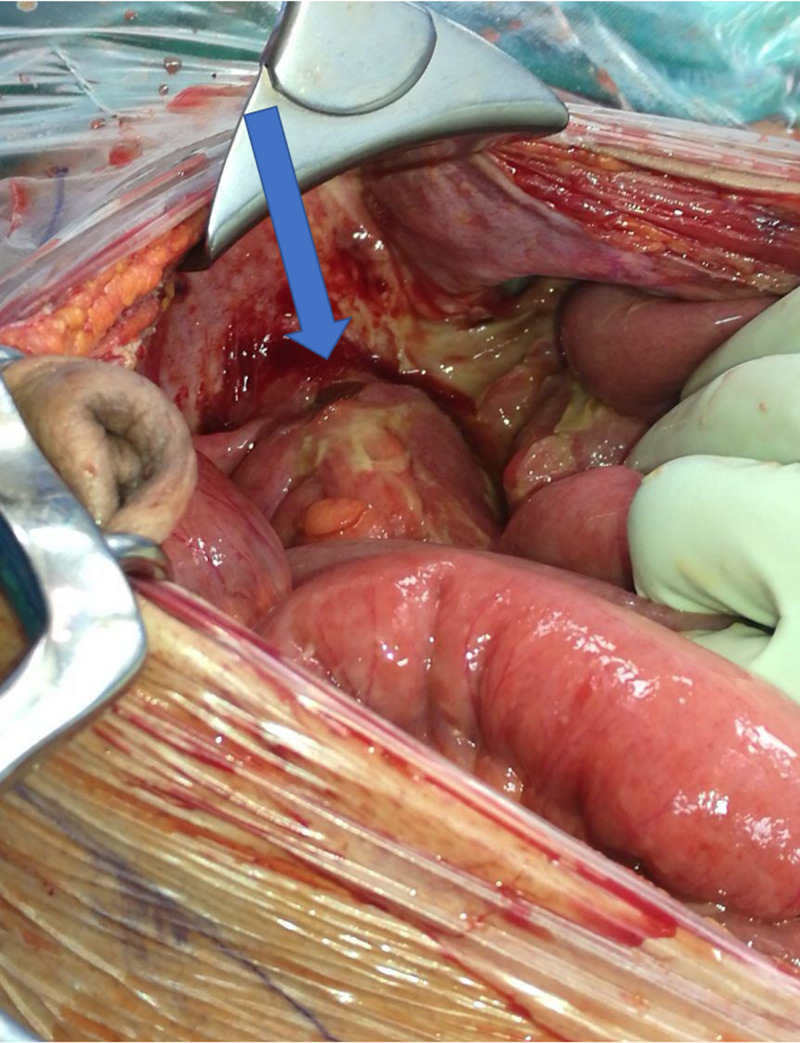
Intraoperative picture of the perforated anterior rectal wall

The patient underwent exploratory laparotomy, which revealed fecal material in the peritoneal cavity. Abdominal exploration showed a perforation at the rectosigmoid junction with the stent exposed (Figure 4). After peritoneal lavage, a loop colostomy was created and a pelvic drain was inserted. The post-operative course was complicated by a pelvic abscess and partial wound dehiscence. Moreover, he developed multiple attacks of rectal bleeding that were controlled through palliative radiotherapy. The total length of hospital stay was two months after which the patient was discharged in a satisfactory condition. Following this admission, he was lost to follow up.

## Discussion

One might expect that combining chemotherapy, bevacizumab, and a self-expanding metal stent would increase the perforation rates. However, the paucity of the literature on this special circumstance limits judgment.

The oncological benefits of bevacizumab in combination with chemotherapy were demonstrated in a phase III trial by Chen et al. [11]. They reported that the addition of bevacizumab to irinotecan/5-fluorouracil/leucovorin as a first-line therapy for metastatic CRC was associated with a significantly increased response rate, progression-free survival, and survival rates.

Multiple studies have demonstrated the risk of perforation in similar conditions, i.e., advanced obstructed rectal cancer combined with metal stent placement, neoadjuvant chemotherapy, and bevacizumab therapy. One study that enrolled all of the patients with advanced CRC who were treated in their institute over an 11-year period, found no significant difference in the incidence of gastrointestinal perforation between the groups that underwent chemotherapy alone and chemotherapy plus bevacizumab, chemotherapy and chemotherapy plus bevacizumab, or chemotherapy alone and no chemotherapy (p = 0.21, p = 0.63, and p = 0.42, respectively) [12]. 

The predisposing factors that are most commonly suggested for bevacizumab-related perforation are peptic ulcer disease, diverticulitis, chemotherapy-induced colitis, a history of abdominal radiation, and abdominal carcinomatosis have been discussed in many articles as independent risk factors; however, there are no associations that have been sufficiently established [13]. Saif et al. [14] reported that the incidence of bowel perforation tends to be higher in candidates with a recent history of colonoscopy or sigmoidoscopy. None of these factors were present in our case except for the patient having a history of bowel obstruction that was treated with a metal stent three weeks prior to the perforation, which may have posed an additional risk of perforation. However, there have been cases reported of bevacizumab-induced bowel perforation in patients that were treated for non-small cell lung cancer despite the absence of evidence of metastatic spread to the abdomen or other predisposing risks for perforation [15].

As in our case, stenting could be an additional risk for patients on bevacizumab. The European Society of Gastrointestinal Endoscopy (ESGE) clinical guidelines provides strong recommendations for the use of self-expanding metal stents as palliative care for advanced obstructed colon cancer, except for in patients using bevacizumab; however, the evidence to substantiate this recommendation is of low quality [16]. 

The timing of systemic chemotherapy administration in relation to stent insertion and the number of cycles may influence the risk of perforation; however, this idea has not yet been well established. A study reported that receiving chemotherapy after stent insertion, regardless of bevacizumab use, raises the incidence of perforation to 11% compared to only 6% in patients who received systemic therapy before the procedure, regardless of bevacizumab use [12]. One reasonable explanation for this is that, the different tumor responses to therapies result in a weakened intestinal wall and subsequent erosion of the applied stent; however, despite its occurrence, this concept could not be applied to therapies after stenting and it requires more justifiable explanations [17].

## Conclusions

There are multiple modalities that can be used to manage obstructed CRC. For patients treated with bevacizumab, the risk of perforation increases, which should be taken into consideration despite it being a rare complication. Stenting on the other hand carries an additional risk of perforation, not to mention that many possible factors could augment or diminish bevacizumab’s perforation risk. This includes interpersonal differences such as demographics, disease onset and course, histological type, and location. In addition, the different treatment modalities, types of chemotherapeutic agents, timing of administration, and the period separating the last dose from the surgical procedure should be considered; however, the scarcity of these conditions has not yet been made clear. Further studies are required to investigate the different associated factors and their possible contributions to similar conditions.
